# Organizational Practices for the Inclusion of People with Disabilities. A Scoping Review

**DOI:** 10.1007/s10926-024-10228-5

**Published:** 2024-07-30

**Authors:** Rik van Berkel, Eric Breit

**Affiliations:** 1 Utrecht School of Governance, Utrecht University, Bijlhouwerstraat 6, 3511ZC Utrecht, Netherlands; 2 Department of Leadership and Organizational Behaviour, BI Norwegian Business School, Nydalsveien 37, 0484 Oslo, Norway

**Keywords:** Inclusion, People with disabilities, Organizational practices, Review, HRM

## Abstract

**Purpose:**

The purpose of the scoping review presented in this article is to map the state-of-the-art and development of empirical research of organizational practices designed to include people with disabilities. It contributes to debates on demand-side approaches in promoting the labour-market participation of people with disabilities.

**Methods:**

A literature search took place in PsychINFO, Web of Science, Sociological Abstracts and Sociological Index. Articles included empirical studies published between 2000 and 2023.

**Results:**

The search resulted in 10,535 unique articles of which 146 were included in the review. Organizational inclusion practices have received increasing attention in academic journals in a variety of research fields. In terms of content two groups of studies can be distinguished: hiring studies and studies focusing on organizational practices aimed at employees with disabilities. Hiring studies include studies analysing relationships between a large range of factors and actual hiring or intention to hire as well as studies of a more exploratory nature. Studies focusing on employees with disabilities look at outcomes of specific organizational practices; the conditions promoting their implementation; or explore practices in organizations employing people with disabilities.

**Discussion:**

Based on the findings of the review three suggestions for future research are discussed: (i) internationally comparative studies; (ii) specific attention to small and medium sized enterprises in studies of inclusion; (iii) systematic reviews as follow-ups to scoping reviews.

**Supplementary Information:**

The online version contains supplementary material available at 10.1007/s10926-024-10228-5.

## Introduction

This article presents the findings of a scoping review of empirical studies of organizational practices for the work inclusion of people with disabilities. Although the labour-market participation of people with disabilities is a core social challenge, studies of the ways that organizations approach their work inclusion are still relatively scarce. This hampers the development and implementation of successful policies, measures and actions to promote sustainable employment for people with disabilities.

Scholars from various backgrounds have drawn attention to the importance of considering the demand-side perspective, a focus on employers and their organizations [1–5]. Already in 1992 Gilbride and Stensrund presented the Demand-side Job Development model, arguing: “(.) this model suggests that we must do more than provide rehabilitation to people with disabilities, we must rehabilitate the environments in which they will work” [2:36]. Attention to employers and the demand-side has also been advocated by social policy scholars, sometimes under the heading of employer engagement [4, 5]: the active involvement of employers in addressing social issues such as the labour-market participation of people with disabilities. Demand-side approaches that actively intervene in organizational practices in order to make organizations more inclusive [5] come closest to rehabilitating the environments in which people with disabilities work.

Against this background the aim of the review presented in this article is to map the state-of-the-art and development of empirical research of organizational practices designed to include people with disabilities. Our review begins by presenting some general characteristics of the articles included in the review and their development between 2000 and 2023. Then we focus on the content of the articles and identify some main lines of research that characterize this area of research. This scoping review contributes to the knowledge base in this area in a number of ways. First, whereas most existing reviews used a more narrow approach and focused on studies that looked at people with specific disabilities [6, 7], addressed specific organizational practices [8–11] or used specific methodological approaches [12–14], our review adopted a broad approach. This enabled us to provide a more general overview of the state-of-the-art of the field of research which may help future researchers in positioning and developing their studies. Secondly, to our knowledge existing reviews paid little attention to developments in the research issue under study. We contribute by also focusing on how the field of research has developed between 2000 and 2023. Thirdly, we will identify some gaps in current research and present some suggestions for future research. Given the broad approach of our review we hope that the discussion of these gaps and suggestions will be relevant for a large audience of scholars of organizational practices for the inclusion of people with disabilities.

## Organizational Practices for the Inclusion of People with Disabilities: Background

Attention to organizational practices in the context of scientific and policy debates about the labour-market participation of people with disabilities is inextricably linked to debates about the concept of disability itself. Instead of being defined in terms of personal attributes disability is increasingly seen as the result of interactions between individuals and their social environment [15, 16]. This interactional concept of disability was the cornerstone of the World Health Organization’s International Classification of Functioning, Disability and Health [17] that defined disability as “an umbrella term for impairments, activity limitations, and participation restrictions. Disability refers to the negative aspects of the interaction between individuals with a health condition (…) and personal and environmental factors (…)” [17:7]. It was also referred to in the Preamble to the United Nations Convention on the Rights of Persons with Disabilities [18]: “disability results from the interaction between persons with impairments and attitudinal and environmental barriers that hinder their full and effective participation in society on an equal basis with others” [18:2]. In the context of promoting the labour-market participation of people with disabilities the focus on their interaction with the environment directed attention to employers and their organizations. Organizational practices became more important focal points of policy interventions. Recruitment, selection and hiring processes received specific attention as they play a key role in organizations’ accessibility for people with disabilities. Policy interventions were introduced addressing these practices: anti-discrimination legislation, quota regulation and positive incentives such as wage subsidies, subsidies for work accommodations or subsidies for job coaching. Nevertheless, inclusion is not ‘finished’ when people with disabilities manage to find employment [19, 20]. Studies have paid attention to issues such as discrimination at the workplace [21, 22], experiences of organizational fairness [23] and disparities between employees with and without disabilities related to, among others, job security and promotion opportunities [24, 25]. These studies point at the importance of addressing inclusion in organizational practices across the employment cycle [26]. For this reason our review included studies addressing pre-hiring organizational practices (recruitment and selection) as well as studies focusing on organizational practices targeted at employees with disabilities (post-hiring).

Attention to organizational practices across the employment cycle has also been promoted in the context of welfare-to-work, activation and rehabilitation policies and interventions. Whereas initially these policies and programmes targeted unemployed, sick and disabled persons by supporting or obliging them to (re-)enter the labour market (supply-side approach), nowadays we see more interventions targeted at employers and their organizations. These demand-side interventions become manifest, among others, in forms of supported employment and services provided to employers by rehabilitation or activation professionals including information and advice on work accommodations, job crafting and job carving, job coaching, et cetera [2–4, 27]. As Chan and colleagues argued, these professionals “must be able to identify demand occupations and work as human resources (HR) consultants to help employers make job modifications and accommodations in order to identify, recruit, and retain qualified persons with disabilities for difficult to fill positions” [3:413].

Summarizing, the study of organizational practices for the inclusion of people with disabilities is of scientific and societal relevance. It provides insight into the role of these practices in encouraging or hampering the labour-market inclusion of people with disabilities both in terms of their job opportunities and in terms of their social and productive inclusion at the workplace. These insights may support societal actors–such as policy makers, rehabilitation and activation professionals, Human Resources staff, trade unions–in their efforts to make organizations more inclusive.

## Method

Scoping reviews are a type of literature review and are often distinguished from systematic reviews. These types of reviews differ in two respects [28]. Systematic reviews focus on well-defined research questions, whereas scoping reviews address broader defined topics. In addition systematic reviews are based on a narrow range of quality assessed studies, whereas scoping reviews have a broader focus and usually do not assess the quality of studies. The characteristics of scoping reviews are related to the objectives of this type of literature review. Arksey and O’Malley [28] listed four possible objectives for undertaking a scoping review: (1) to examine the extent, nature and range of research activity; (2) to determine the value of undertaking a full systematic review; (3) to summarize and disseminate research findings; and (4) to identify research gaps in the existing literature. The first of these objectives comes closest to the primary objective of our literature review. Because of this, and because our research topic is broad rather than narrow (for example, the impact of a specific organizational practice on the performance of employees with disabilities) we consider the scoping review appropriate for our literature review.

For reporting the scoping review we were guided by the PRISMA-ScR checklist. PRISMA-ScR stands for Preferred Reporting Items for Systematic reviews and Meta-Analyses: extension for Scoping Reviews [29]. It provides an overview of items to be reported in scoping reviews. A significant part of these items concerns guidelines for reporting the methods used during the review.

### Selection and Screening

The aim presented in the introduction guided the development of a search string for the identification of relevant studies through multiple database searches. The search string was divided into three groups of search terms. The first group was related to the target group and included terms such as disab* and impair*. The second group related to organizational practices and included search terms such as HRM practice*, job accommodation*, et cetera. The third group related to employment outcomes. Examples of terms in this group included job and employ*. The three groups were combined (‘Target group’ AND ‘Organizational practices’ AND ‘Employment outcome’) so that each article had to contain at least one search term from each of the three groups either in its title or in its abstract. As additional selection criteria we focused on English language articles in peer-reviewed journals in the period 2000–2023. Reducing the time span to the twenty-first century was done for pragmatic reasons. Nevertheless, in hindsight (see the results section) one could argue that the early 2000s represented the early days of studies of organizational practices for the inclusion of people with disabilities, which was then followed by a period of gradual expansion of research into the topic. Furthermore, we searched for articles presenting results of empirical studies and therefore excluded reviews. Four electronic databases were searched: PsychINFO, Web of Science, Sociological Abstracts and Sociological Index. As the initial search yielded a substantial number of articles a NOT group was added to the search string to exclude irrelevant articles. Database searches returned 12,538 articles, reduced to 10,535 after removal of duplicates.

After the initial search additional *post-hoc* inclusion/exclusion criteria were developed. One additional *post-hoc* exclusion criterion needs specific attention here. A significant number of articles in our sample explicitly and exclusively focused on Return-to-Work processes and interventions. Our review was part of a larger project focusing on barriers and opportunities for people with disabilities in entering and maintaining employment. It was our assumption that Return-to-Work interventions mainly focus on another target group: non-disabled employees who become sick or disabled while in employment. Because of that and because we needed to reduce the number of articles we decided to exclude Return-to-Work studies. In hindsight this decision may be questioned. Both non-disabled and disabled employees may require Return-to-Work processes and these processes may play an important role in the sustainability of their employment and work inclusion [30].

In this step of the selection process titles and abstracts were screened using the review application Rayyan. Rayyan is a research collaboration platform. Collaborating researchers use it to screen articles independently and to compare results after screening is completed. For our review articles were divided between pairs of researchers. Each member of a research pair reviewed the same abstracts and titles independently and decided to include or exclude the studies. After screening the pairs resolved conflicting decisions of inclusion and exclusion. Cases of uncertainty were solved in the full team of five researchers. In cases where title or abstract provided insufficient information to reach an exclusion or inclusion decision the full article was screened. In addition, articles were excluded if they were published in a journal without an impact factor. This criterion was used to further reduce the number of articles. This process resulted in a list of 146 articles. For a schematic overview of the selection process and the full search string, see Online Resource 1.

### Data Extraction and Analysis

A thorough reading of the 146 articles took place with the aim of extracting two types of data. The first type of data referred to general characteristics of the studies reported in the articles: year of publication, journal of publication, country/countries where data collection took place, research methodology (quantitative methods, qualitative methods or a mix of methods), research objects and size of organizations where the studies took place (Small and Medium-sized Enterprises (“SMEs”) or large organizations). We also looked at whether or not the studies specified the types of disabilities of the people they focused on. In the former case we assigned the studies to one of the following three groups: studies that focused on people with physical or sensory disabilities; studies focusing on people with mental, intellectual or developmental disabilities; and studies focusing on mixes of these two groups. We did this categorization for all articles, also those that did not have people with disabilities as respondents but studied other organizational actors’ experiences with or perceptions of working with people with disabilities. All these data were entered into SPSS for further analysis. In order to get an indication of how these characteristics developed over time we compared the articles published during the first four years of the twenty-first century (2000–2003) with articles published in the four most recent years (2020–2023). We used a four year period as during the first years of the twenty-first century the number of publications was small.

The second type of data extracted from the articles referred to content characteristics of the studies. First, the articles were divided into two main groups, reflecting the distinction between *pre-hiring* and *post-hiring* organizational practices discussed above. The first group we distinguished were studies focusing on the hiring (including recruitment and selection) of people with disabilities. The second group of articles addressed organizational practices for the inclusion of people with disabilities who successfully passed the hiring process, i.e. employees with disabilities. Eight articles were placed in both groups as they focused on pre-hiring as well as post-hiring practices. Secondly, we explored whether more refined categorizations could be made within each of the two main groups.

Online Resource 2 provides an overview of all articles included in this review. Online Resource 3 presents some core characteristics of each of the articles.

## Results

### General Characteristics of the Studies

#### The Number of Articles

Half of the 146 articles in our review were published in the 2020–2023 period. Compared to the 2000–2003 period, in which nine articles were published, the number of articles published in 2020–2023 was eight times as high. This growth does not necessarily imply that the topic of practices in organizations for the inclusion of people with disabilities has been receiving more scientific attention. It may merely reflect the growth of academic output, i.e. articles in scientific journals, in general. Therefore we looked at two other growth figures using data from Scimago Journal & Country Rank, a publicly available portal that includes the journals and country scientific indicators developed from the information contained in the Scopus® database [31]*.* First, we looked at the increase of number of journal articles in the social sciences (subcategory ‘social sciences (miscellaneous)’) in the 2000–2022 period (2022 was the most recent year for which data was available). In 2022, the number of publications was close to five times higher than in 2000. Secondly, we took a more focused approach by looking at the growth in number of articles in the six journals that published most articles included in our review: *Disability and Rehabilitation* (7 articles)*, Disability & Society* (13 articles), *International Journal of Human Resource Management* (7 articles), *Journal of Occupational Rehabilitation* (12 articles), *Rehabilitation Counselling Bulletin* (12 articles) and *Work* (7 articles)*.* Comparing the number of articles published in these journals during the three years before 2023 with the three years before 2003 revealed a growth factor of 3. This data supports the conclusion that the research topic of our review study is receiving increasing attention, both in the social sciences more generally and in journals relevant for the research topic.

#### The Journals

The articles in our review were published in 63 academic journals. Organizational inclusion practices are being studied from a variety of perspectives and are an interesting topic for multidisciplinary research. To analyse this issue further we categorized the journals based on their titles and main topic of interest. This resulted in six categories: five research areas (Social policy & Social work; Organization/Work/Human Resource Management; Rehabilitation; Disability; Health & Medicine) and one category ‘Other’ (for a full overview see Online Resource 4). The Rehabilitation category contained most articles (34%). Disability (21%) and Organization/Work/HRM (27%) are relevant for the topic of our review as well. The Social policy/Social work category and Health & Medicine journals published 5% of articles each. All articles from the 2000 to 2003 period were published in Rehabilitation or Disability journals. In the 2020–2023 period the other research areas and specifically Organization/Work/HRM published on the topic as well.

#### Countries of Data Collection

Most of the studies took place in North America (USA and Canada): 39%. 36% of the studies were conducted in European countries. Far smaller proportions of studies took place in the other continents. Here again we see developments over time. The 2000–2003 period was dominated by North American studies (78%). In the 2020–2023 period the largest proportion of studies came from European countries (48%). Almost all studies took place in a single country. International comparative studies are scarce: only six studies compared countries, two within Europe [32, 33] and four across continents [34–37].

#### Methodology

Qualitative and quantitative methods balance each other out in the studies. Qualitative methods mainly involved interviews, although other methods were used as well: for example, participant observation and informal conversations. Surveys were the predominantly used quantitative method. Some studies used secondary analysis of existing survey or register data, some used (semi)experimental designs. Over time only minor differences in the use of qualitative and quantitative methods were observed.

#### Research Objects

One third of the studies used a multi-actor approach, focusing research on two or more groups of actors, for example, employees with and without disabilities or disabled employees and supervisors. Half of all studies focused on employers/owners or staff with HR responsibilities in organizations. A similar proportion of studies had people with disabilities as research objects. In a quarter of the studies non-disabled employees (often but not always with disabled people as colleagues) or supervisors were respondents. In a small group of studies rehabilitation or reintegration professionals (vocational/occupational rehabilitation staff, job coaches, employment specialists, et cetera) were research objects. Comparing the 2000–2003 and the 2020–2023 periods most studies in the first period focused on employers/owners and HR staff. In the second period people with disabilities were the dominant research group.

#### People with Disabilities

We also looked at the groups of people with disabilities that the studies focused on. The largest group of studies (37%) did not specify the type of disabilities. 20% of the studies focused on people with physical disabilities, 25% on people with mental, intellectual or developmental disabilities. 18% of the studies looked at people from both groups. Comparing the two periods we see that in the most recent period a larger proportion of studies did not specify the type of disabilities.

#### Attention to SMEs

Finally we looked at the size of organizations that were studied. In particular, we were interested in the question whether studies paid attention to SMEs. One fifth of the studies paid specific attention to SMEs, focusing on SMEs exclusively or comparing large organizations and SMEs (sometimes through ‘size’ as the unit of comparison). Studies comparing large organizations and SMEs often pointed at the lack of facilitators for SMEs compared to large organizations such as lack of resources, professional HR staff, or formal policies. The other studies used samples including SMEs but did not analyse the role of size; focused on large organizations only; or did not mention the size of the organizations where the study took place. Comparing the two periods we see that specific attention to SMEs has increased.

Table 1 summarizes the main findings concerning general characteristics of the articles.

**Table 1:** General characteristics of the studies in the review

	All articles	Articles published from 2000 to 2003	Articles published from 2020 to 2023
Number of articles	146	9	73
*Journal categories (%)*			
Rehabilitation	34	44	29
Disability	21	56	20
Social policy/social work	5	0	7
Organization/Work/HRM	27	0	27
Health/Medicine	5	0	7
Other	8	0	10
*Methods (%)*			
Qualitative	46	44	49
Quantitative	50	56	47
Mix	4	0	4
*Continent (%)*			
Africa	3	0	4
Asia	8	0	5
Australia/Oceania	10	11	11
Europe	35	11	48
North America	39	78	29
South America	2	0	1
Mix	3	0	1
*Research objects (%)*			
Employers/owners, HR staff	51	67	47
People with disabilities	50	44	56
Colleagues, supervisors	25	11	31
Professionals, service providers	11	33	11
*Types of disability (%)*			
Not specified	37	22	37
Physical	20	22	21
Mental, intellectual, developmental	25	22	21
Mix	18	33	22
*Other characteristics (%)*			
Multi-actor approach	34	33	38
International comparative	4	0	3
Attention to SMEs	21	0	12

### Content of the Studies

Of the articles in our review 29% belonged to the group of hiring studies. A majority of studies (66%) belonged to the group of studies addressing organizational practices for employees with disabilities. A small group of studies (5%) belonged to both groups and studied a broad range of organizational practices (including recruitment and selection) developed in organizations employing people with disabilities. Over time only minor changes in the distribution of articles over the groups took place. Hiring studies are somewhat more likely to be published in Disability journals, which is probably due to the fact that issues of labour-market discrimination and inequality of employment opportunities are more prominent in this group of journals. Rehabilitation and Organization/Work/HRM journals are somewhat more likely to publish articles in the second group.

In hiring studies employers/owners/HR staff are the main group of research objects: 88% had employers/owners/HR staff as respondents. Evidently these actors are most strongly involved in hiring policies and practices. The picture looks different when we look at the studies focused on organizational practices for employees with disabilities. Of the articles in this group (several studies used a multi-actor approach) two thirds had people with disabilities as their research objects and one third had non-disabled colleagues or supervisors as respondents. Employers/owners/HR staff were respondents in one third of these studies. Compared to hiring studies these studies not only have a less dominant management perspective but also pay considerable attention to perceptions and experiences of employees with disabilities.

An overarching issue arising from analysing the content of the studies is whether they focused on general organizational or human resource management (HRM) practices, i.e. practices targeted at all employees or job applicants; or looked at practices specifically designed to promote inclusion of people with disabilities. In hiring studies the latter perspective is most dominant. Most studies focus on specific selection and recruitment practices designed to attract people with disabilities such as providing disability training for recruiters, collaborating with vocational rehabilitation agencies or public employment services or characteristics of selection processes. Only a few hiring studies focus on general organizational practices such as health and wellness programmes for employees or retention practices. The picture is more mixed in the second group of studies. Disability specific practices that the studies analysed include, for example, forms of support provided to employees with disabilities or disability management practices. General organizational practices being studied are supervisor and leadership styles, job security, job flexibility and telework. Of course, the distinction between general and specific practices is not black and white. For example, organizations can provide work accommodations for people with disabilities specifically but they can offer accommodations to all employees as well.

Below we will analyse the content of articles in both groups in more detail (see Online Resource 3 for an overview of our categorization of articles in groups).

#### Hiring Studies

Hiring studies have in common that they are interested in fathoming the hiring process and in identifying factors that hamper (barriers) or support (facilitators) organizations to employ people with disabilities. Analysis of the hiring studies resulted in the distinction of three subgroups. Studies in the first subgroup identified factors that are related to actual hiring. These studies measured actual hiring in various ways such as currently employing people with disabilities or not; recently hired people with disabilities or not; or percentage of employees with disabilities in organizations. A majority of these studies used quantitative methods. The variety of factors being studied is considerable. Factors include ‘hard’ organizational characteristics such as size, industry and sector, but also ‘soft’ factors such as attitudes towards people with disabilities and their employability; collaboration with external rehabilitation or employer services; written policies and procedures; national policies and policy instruments; et cetera.

The second subgroup of hiring studies did not look at actual hiring but at what are sometimes seen as proxies or antecedents of actual hiring such as: likelihood or intention to hire; actively looking for persons with disabilities when looking for new employees; or inviting people with disabilities for job interviews. It is useful to distinguish between these two subgroups as studies have shown that these proxies are not necessarily good predictors of actual hiring [35, 38]. Most studies in this subgroup were quantitative.

The third and largest subgroup consisted of hiring studies of a more exploratory nature. They explored what factors or how specific factors have an impact on hiring processes without actually testing the relationship between factors and hiring. Many of these studies focused on employers’ perspectives or perceptions related to factors such as framing of disability, presentation and disclosure of disability in job interviews, types of disability and expectations of disabled people’s performance and employability. These studies are more varied in their methods than the other subgroups.

#### Studies of Practices Addressing Inclusion of Employees with Disabilities

The second group of studies focused on organizational practices addressing the inclusion of employees with disabilities and took place in organizations that employed people with disabilities. Here again three subgroups of studies can be distinguished.

The first and largest subgroup studied outcomes of organizational practices. The variety of organizational practices addressed is considerable. In an attempt to categorize these practices we came to the following core groups of practices: job development and accommodations; workplace relations and organizational culture; support for employees with disabilities; training and development of employees with disabilities; leadership; work conditions; participation; performance management; and general HRM characteristics (such as fairness and supportiveness). Variation also characterized the outcomes the studies analysed. Four types of outcomes were most frequently studied: (i) sustainable employment (operationalized, among others, in terms of retention, job tenure, turnover intention); (ii) health and well-being of employees with disabilities (operationalized, among others, in terms of job satisfaction, positive experiences); (iii) performance and productivity (operationalized, among others, in terms of job performance, wages, employability); and (iv) workplace inclusion (operationalized, among others, in terms of social or worksite integration).

The second subgroup focused on conditions conducive to the implementation of organizational practices seen as contributing to inclusion. The conditions that the studies looked at include firm size, type or severity of disability, national policies as well as attitudes or moral beliefs. Making accommodations/adjustments and providing support were the most frequently studied organizational practices in this subgroup.

The third subgroup in this group of studies is more exploratory. These studies focused on organizational practices in organizations employing people with disabilities without analysing specific outcomes or conditions, which was the case in the first two subgroups. Some studies aimed to identify promising practices in organizations employing people with disabilities but most studies had a broader purpose. They also looked at barriers and challenges where the inclusion of employees with disabilities is concerned. The organizational practices discussed in the studies covered most of the core groups of practices that were found in the first subgroup.

## Discussion and Suggestions for Future Research

We have seen that the research topic of inclusive organizational practices is being studied all over the world. However, international comparative studies are still scarce. Hiring studies can function as an example to illustrate why this can be considered a gap in the literature [39]. As was mentioned before many countries have introduced incentives for employers (anti-discrimination or quota regulations, subsidies and services for employers) to encourage them to hire people with disabilities. Although several hiring studies pointed at the impact of policies on barriers and facilitators for hiring people with disabilities, international comparative studies could provide more in-depth insights into the effects of various policy interventions and how they influence hiring decisions in organizations. This way, internationally comparative policy studies could be linked to studies of organizational hiring practices and decisions. A similar argument can be developed with respect to other country-specific factors that may facilitate or hamper organizations to become more inclusive, such as cultural influences on perceptions of people with disabilities [32] and the role of industrial relations regimes in the employment of people with disabilities [40].

The second gap we identify considers attention to SMEs. According to the World Bank, SMEs represent 90% of businesses and 50% of employment worldwide (see World Bank SME Finance). As our review revealed, SMEs received relatively little specific attention up until now. Available studies comparing large organizations and SMEs often pointed at the lack of resources of SMEs. Less attention was paid to the specific characteristics (such as family-like environment, informality and less bureaucracy) and potential advantages of SMEs compared to large organizations [41–43]. What is more, several authors have argued that researchers should be careful in treating large organizations as models for how inclusion in SMEs could be promoted [41, 44]. In our view these arguments provide a strong case for future research of SMEs.

One of the possible purposes of scoping reviews is to explore the possibilities for systematic reviews. Here we present two suggestions for systematic reviews that specifically focus on studies looking at outcomes of organizational practices for employees with disabilities. A systematic review of studies published between 1990 and 2020 and focusing on organizational practices and their outcomes [45] concluded that most studies focused on hiring and that studies of *post-hiring* organizational practices were scarce. As our review showed this situation has improved considerably in recent years, which makes systematic reviews of these practices and their outcomes possible and desirable. The fact that existing studies have used various groups of respondents is of added value as it makes it possible to study outcomes from various perspectives. The second suggestion concerns the distinction we made between general organizational practices and practices specifically addressing the needs of people with disabilities. A systematic review could address the issue that was also mentioned in the review referred to above [45], namely to what degree general organizational practices suffice to cater to the needs of people with disabilities and where specific practices are necessary. Where general organizational practices are concerned, multi-actor studies focusing on experiences and perceptions of employees with and without disabilities are particularly useful. The issue of general versus targeted or tailor-made approaches could also be expanded, for example, when we want to look at the inclusion of vulnerable groups more generally versus people with disabilities specifically [46] or when we look at the inclusion of people with disabilities in general or persons with specific disabilities in particular [47].

The diversity of perspectives from which the research topic of organizational practices for people with disabilities is being studied will enrich the results that can be derived from these systematic reviews. At the same time, this diversity will also provide a challenge for scholars interested in conducting such reviews. Discourses, concepts and theoretical frameworks differ across the various perspectives which makes summarizing and integrating findings complex. We hope that our review will inspire scholars to accept this challenge.

## Supplementary Information

Below is the link to the electronic supplementary material.

Supplementary file1 (PDF 112 kb)

Supplementary file2 (PDF 372 kb)

Supplementary file3 (PDF 183 kb)

Supplementary file4 (PDF 126 kb)

## Data Availability

No datasets were generated or analysed during the current study.

## References

[1] Lengnick-Hall M, Gaunt P, Kulkarni M. Overlooked and underutilized: people with disabilities are an untapped human resource. Hum Resour Manag. 2008;47(2):255–73. 10.1002/hrm.20211.

[2] Gilbride D, Stensrund R. Demand-side job development: a model for the 1990s. J Rehabil. 1992;58(4):34–40.

[3] Chan F, Strauser D, Maher P, Lee E, Jones R, Johnson E. Demand-side factors related to employment of people with disabilities: a survey of employers in the Midwest region of the United States. J Occup Rehabil. 2010;20:412–9. 10.1007/s10926-010-9252-6. 20602153 10.1007/s10926-010-9252-6

[4] Ingold J, Stuart M. The demand-side of active labour market Policies: a regional study of employer engagement in the Work Programme. J Soc Pol. 2015;44(3):443–62. 10.1017/S0047279414000890.

[5] Ingold J, McGurk P, editors. Employer engagement: Making active labour market policies work. Bristol: Bristol University Press; 2023.

[6] Ellenkamp J, Brouwers E, Embregts P, Joosen M, Van Weeghel J. Work environment-related factors in obtaining and maintaining work in a competitive employment setting for employees with intellectual disabilities: a systematic review. J Occup Rehabil. 2016;26(1):56–69. 10.1007/s10926-015-9586-1. 26112400 10.1007/s10926-015-9586-1 PMC4749651

[7] Padkapayeva K, Posen A, Yazdani A, Buettgen A, Mahood Q, Tompa E. Workplace accommodations for persons with physical disabilities: evidence synthesis of the peer-reviewed literature. Disabil Rehabil. 2017;39(21):2134–47. 10.1080/09638288.2016.1224276. 27936968 10.1080/09638288.2016.1224276

[8] Gewurtz R, Langan S, Shand D. Hiring people with disabilities: a scoping review. Work. 2016;54(1):135–48. 10.3233/WOR-162265. 26967030 10.3233/WOR-162265

[9] Nagtegaal R, De Boer N, Van Berkel R, Derks B, Tummers L. Why do employers (fail to) hire people with disabilities? A systematic review of capabilities, opportunities and motivations. J Occup Rehabil. 2023;33(2):329–40. 10.1007/s10926-022-10076-1. 36689057 10.1007/s10926-022-10076-1 PMC10172218

[10] Nevala N, Pehkonen I, Koskela I, Ruusuvuori J, Anttila H. Workplace accommodation among persons with disabilities: a systematic review of its effectiveness and barriers or facilitators. J Occup Rehabil. 2015;25(2):432–48. 10.1007/s10926-014-9548-z.

[11] Chen X, Wu J, Grenawalt T, Mpofu N, Chan F, Tansey T. Employer practices for customized training for onboarding of people with disabilities. Rehabil Res Pol Educ. 2023;37(1):10–23. 10.1891/RE-22-13.

[12] Heera S, Devi A. Employers’ perspective towards people with disabilities. A review of the literature. S E Asian J Manage. 2016;10(1):54–74. 10.21002/seam.v10i1.5960.

[13] Kersten A, Van Woerkom M, Geuskens G, Blonk R. Organizational policies and practices for the inclusion of vulnerable workers: a scoping review of the employer’s perspective. J Occup Rehabil. 2023;33(2):245–66. 10.1007/s10926-022-10067-2. 36083361 10.1007/s10926-022-10067-2 PMC9461424

[14] Lindsay S, Cagliostro E, Albarico M, Mortaji N, Karon L. A systematic review of the benefits of hiring people with disabilities. J Occup Rehabil. 2018;28(4):634–55. 10.1007/s10926-018-9756-z. 29392591 10.1007/s10926-018-9756-z

[15] Vornholt K, Villotti P, Muschalla B, Bauer J, Colella A, Zijlstra F, Van Ruitenbeek G, Uitdewilligen S, Corbière M. Disability and employment – overview and highlights. Eur J Work Organ Psy. 2018;27(1):40–55. 10.1080/1359432X.2017.1387536.

[16] Williams J, Mavin S. Disability as constructed difference: a literature review and research agenda for management and organization studies. Int J Manag Rev. 2012;14(2):159–79. 10.1111/J.1468-2370.2012.00329.X.

[17] World Health Organization. World report on disability: Summary. Geneva: World Report on Disability (who.int); 2011.

[18] United Nations. Convention on the rights of persons with disabilities. Convention on the Rights of Persons with Disabilities | OHCHR; 2006

[19] Mor BM. Beyond affirmative action. Admin Soc Work. 1999;23(3–4):47–68. 10.1300/J147v23n03_04.

[20] Shore L, Cleveland J, Sanchez D. Inclusive workplaces: a review and model. Hum Resour Manage R. 2018;28(2):176–89. 10.1016/j.hrmr.2017.07.003.

[21] Mousa M, Samara G. The institutional limitations of emancipation: the inclusion of disabled employees in the Egyptian public context post COVID-19. Int J Public Admin. 2022;46(13):939–50. 10.1080/01900692.2022.2049815.

[22] Eissenstat S, Lee Y, Hong S. An examination of barriers and facilitators of job satisfaction and job tenure among persons with disability in South Korea. Rehabil Couns Bull. 2022;65(4):310–21. 10.1177/00343552211006767.

[23] Chordiya R. Organizational inclusion and turnover intentions of federal employees with disabilities. Rev Public Pers Admin. 2020;42(1):60–87. 10.1177/0734371X20942305.

[24] Schur L, Han K, Kim A, Ameri M, Blanck P, Kruse D. Disability at work: a look back and forward. J Occup Rehabil. 2017;27(4):482–97. 10.1007/s10926-017-9739-5. 29110160 10.1007/s10926-017-9739-5

[25] Schur L, Kruse D, Blasi J, Blanck P. Is disability disabling in all workplaces? Workplace disparities and corporate culture. Ind Relat. 2009;48(3):381–411. 10.1111/j.1468-232X.2009.00565.x.

[26] Bonaccio S, Connelly C, Gellatly I, Jetha A, Ginis K. The participation of people with disabilities in the workplace across the employment cycle: employer concerns and research evidence. J Bus Psychol. 2020;35(2):13–58. 10.1007/s10869-018-9602-5. 10.1007/s10869-018-9602-5 PMC7114957 32269418

[27] Wu J, Iwanaga K, Grenawalt T, Mpofu N, Chan F, Lee B, Tanseye T. Employer practices for integrating people with disabilities into the workplace: a scoping review. Rehab Res Pol Educ. 2023;37(1):60–80. 10.1891/RE-21-25.

[28] Arksey H, O’Malley L. Scoping studies: towards a methodological framework. Int J of Soc Res Method. 2005;8(1):19–32. 10.1080/1364557032000119616.

[29] Tricco A, Lillie E, Zarin W, O’Brien K, Colquhoun H, Levac D, Moher D, Peters M, Horsley T, Weeks L. PRISMA extension for scoping reviews (PRISMA-ScR): checklist and explanation. Ann Intern Med. 2018;169(7):467–73. 10.7326/M18-0850. 30178033 10.7326/M18-0850

[30] Jansen J, Van Ooijen R, Koning P, Boot C, Brouwer S. The role of the employer in supporting work participation of workers with disabilities: a systematic literature review using an interdisciplinary approach. J Occup Rehab. 2021;31(4):916–49. 10.1007/s10926-021-09978-3. 10.1007/s10926-021-09978-3 PMC8558169 33978875

[31] SCImago. SJR — SCImago Journal & Country Rank [Portal]. Retrieved 12 Feb 2024. http://www.scimagojr.com

[32] Grzeskowiak A, Zaluska U, Kwiatkowska-Ciotucha D, Kozyra C. People with disabilities in the workplace: results of a survey conducted among Polish and Finnish employers. Int J Env Res Public He. 2021;18(20):10934. 10.3390/ijerph182010934. 10.3390/ijerph182010934 PMC8535350 34682681

[33] Moody L, Saunders J, Leber M, Wojcik-Augustyniak W, Szajczyk M, Rebernik N. An exploratory study of barriers to inclusion in the European workplace. Disabil Rehabil. 2017;39(20):2047–54. 10.1080/09638288.2016.1217072. 27665671 10.1080/09638288.2016.1217072

[34] Kosyluk K, Corrigan R. Employer stigma as a mediator between past and future hiring behavior. Rehabil Couns Bull. 2014;57(2):102–8. 10.1177/0034355213496284.

[35] Østerud K, Vedeler J. Disability and regulatory approaches to employer engagement: cross-national challenges in bridging the gap between motivation and hiring practice. Soc Pol Society. 2022;23(1):124–40. 10.1017/S1474746422000021.

[36] Vedeler J, Schreuer N. Policy in action: stories on the workplace accommodation process. J Disabil Pol Stu. 2011;22(2):95–105. 10.1177/1044207310395942.

[37] Villotti P, Corbiere M, Fossey E, Fraccaroli F, Lecomte T, Harvey C. Work accommodations and natural supports for employees with severe mental illness in social businesses: an international comparison. Community Ment Hlt J. 2017;53(7):864–70. 10.1007/s10597-016-0068-5. 10.1007/s10597-016-0068-5 27913895

[38] Araten-Bergman T. Managers’ hiring intentions and the actual hiring of qualified workers with disabilities. Int J Hum Resour Manag. 2016;27(14):1510–30. 10.1080/09585192.2015.1128466.

[39] Beatty J, Baldridge D, Boehm S, Kulkarni M, Colella A. On the treatment of persons with disabilities in organizations: a review and research agenda. Hum Resour Manage. 2019;58(2):119–37. 10.1002/hrm.21940.

[40] Foster D, Masso M, Osila L. Work accommodations and sustainable working: the role of social partners and industrial relations in the employment of disabled and older people in Estonia, Hungary and Poland. Eur J Ind Relat. 2021;27(2):149–65. 10.1177/09596801221075077.

[41] Bacon N, Hoque K. The treatment of disabled individuals in small, medium-sized, and large firms. Hum Resour Manag. 2022;61(2):137–56. 10.1002/hrm.22084.

[42] Ulstein J. The impact of employer characteristics on sustaining employment for workers with reduced capacity: evidence from Norwegian register data. Soc Pol Soc. 2023. 10.1017/S1474746423000027.

[43] Ameri M, Kurtzberg T. Small empires: how equipped are small business owners to hire people with disabilities? J Occup Rehabil. 2023. 10.1007/s10926-023-10152-0. 38038802 10.1007/s10926-023-10152-0

[44] Munsell E, Kudla A, Su H, Wong J, Crown D, Capraro P, Trierweiler R, Park M, Heinemann A. Employers’ perceptions of challenges and strategies in hiring, retaining, and promoting employees with physical disabilities. Rehabil Couns Bull. 2022;67(3):177–89. 10.1177/00343552221130304.

[45] Schloemer-Jarvis A, Bader B, Böhm S. The role of human resource practices for including persons with disabilities in the workforce: a systematic literature review. Int J Hum Resour Manag. 2022;33(1):45–98. 10.1080/09585192.2021.1996433.

[46] Kersten A, van Woerkom M, Geuskens G, Blonk R. A classification of human resource management bundles for the inclusion of vulnerable workers. Work. 2024. 10.3233/WOR-230314. 38217565 10.3233/WOR-230314 PMC11492041

[47] Gignac M, Shahidi F, Jetha A, Kristman V, Bowring J, Cameron J, Tonima S, Ibrahim S. Impacts of the COVID-19 pandemic on health, financial worries, and perceived organizational support among people living with disabilities in Canada. Disabil Hlth J. 2021. 10.1016/j.dhjo.2021.101161. 10.1016/j.dhjo.2021.101161 PMC8436082 34246591

