# Supplementary material for: Organizational Practices for the Inclusion of People with Disabilities. A Scoping Review
Source: J Occup Rehabil. 2024 Jul 30;35(3):469–78. doi: 10.1007/s10926-024-10228-5 (PMC12361267; doi:10.1007/s10926-024-10228-5)
Supplement: Supplementary file 1 — Supplementary file1 (PDF 112 kb) [file 10926_2024_10228_MOESM1_ESM.pdf]

ORGANIZATIONAL PRACTICES FOR THE INCLUSION OF PEOPLE WITH DISABILITIES
Journal of Occupational Rehabilitation
Rik van Berkel, Eric Breit
r.vanberkel@uu.nl

Appendix 1. Scoping review flow chart

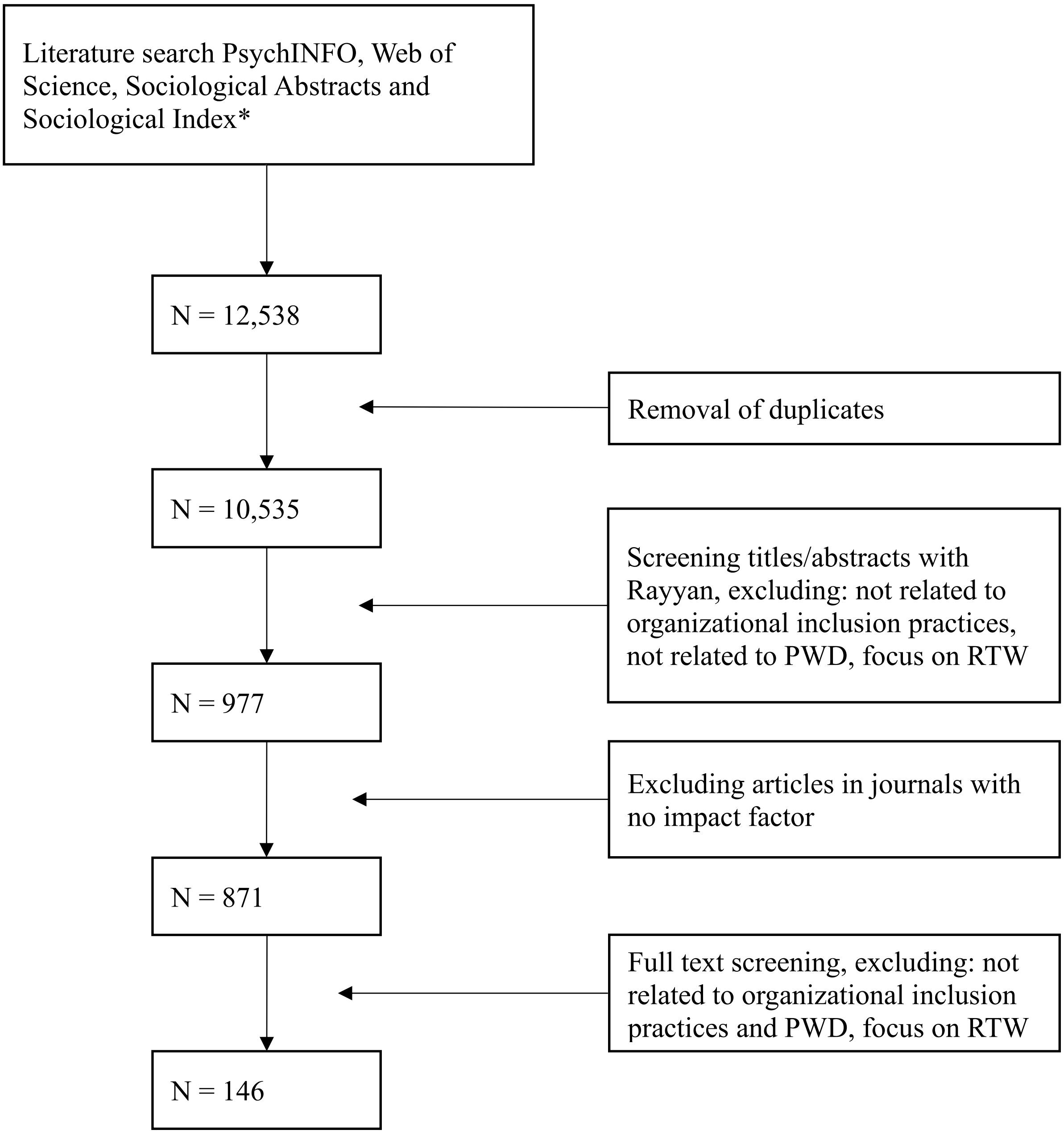

*Full search string:
Target group: disab* OR impair* OR handicap*
AND
Workplace practice: job accommodation* OR job adjustment* OR job modification* OR workplace accommodation* OR workplace adjustment* OR workplace modification* OR

workplace train* OR job coach* OR intervention* OR HRM polic* OR HRM practice* OR support* OR vocation* OR rehabilitation*

AND

Employment outcome: employ* OR work* OR job OR vocation* OR occupation* OR labo?r-market participation OR competitive employ* OR supported employ* OR incl* OR diversity OR integration

AND

NOT: child* OR age?ing OR student* OR gerontology* OR pregnan*
