# Supplementary material for: Organizational Practices for the Inclusion of People with Disabilities. A Scoping Review
Source: J Occup Rehabil. 2024 Jul 30;35(3):469–78. doi: 10.1007/s10926-024-10228-5 (PMC12361267; doi:10.1007/s10926-024-10228-5)
Supplement: Supplementary file 2 — Supplementary file2 (PDF 372 kb) [file 10926_2024_10228_MOESM2_ESM.pdf]

ORGANIZATIONAL PRACTICES FOR THE INCLUSION OF PEOPLE WITH DISABILITIES

Journal of Occupational Rehabilitation

Rik van Berkel, Eric Breit
r.vanberkel@uu.nl

Appendix 2. Articles in review

Ameri M, Kruse D, Park S, Rodgers Y, Schur L. Telework during the pandemic: Patterns, challenges, and opportunities for people with disabilities. Disabil Hlth J. 2023:16(2):101406. https://doi.org/10.1016/j.dhjo.2022.101406
Ameri M., Kurtzberg T. Small empires: How equipped are small business owners to hire people with disabilities? J Occup Rehabil. 2023. https://doi.org/10.1007/s10926-023-10152-0
Araten-Bergman T. Managers' hiring intentions and the actual hiring of qualified workers with disabilities. Int J Hum Resour Manag. 2016:27(14):1510-30. https://doi.org/10.1080/09585192.2015.1128466
Baldwin, S., Costley, D. & Warren, A. Employment activities and experiences of adults with high-functioning autism and Asperger's disorder. J Autism Dev Disord. 2014:44(10):2440-9. https://doi.org/10.1007/s10803-014-2112-z
Banks B, Charleston S, Grossi T, Mank D. Workplace supports, job performance, and integration outcomes for people with psychiatric disabilities. Psychiatr Rehabil J. 2001:24(4):389-97. https://doi.org/10.1037/h0095066
Bartram T, Cavanagh J, Meacham H, Pariona-Cabrera P. Re-calibrating HRM to improve the work experiences for workers with intellectual disability. Asia Pacific J Hum Resour. 2021:59(1):63-83. https://doi.org/10.1111/1744-7941.12230
Becerra M, Montanero M, Lucero M. Graphic support resources for workers with intellectual disability engaged in office tasks: a comparison with verbal instructions from a work mate. Disabil Rehabil. 2018:40(4):435-43. https://doi.org/10.1080/09638288.2016.1258739
Berre, S. Exploring disability disadvantage in hiring: A factorial survey among Norwegian employers. Work Employ Soc. 2023. https://doi.org/10.1177/09500170231175776
Bishop M, Stenhoff D, Bradley K, Allen C. The differential effect of epilepsy labels on employer perceptions: Report of a pilot study. Epilepsy Behav. 2007:11(3):351-6. https://doi.org/10.1016/j.yebeh.2007.06.010
Bjørnshagen W. Do large employers discriminate less? An exploration of company size variation in disability discrimination based on data from two field experiments. Work Occupation. 2022:49(4):483-511. https://doi.org/10.1177/07308884221126877
Bjornshagen V, Ugreninov E. Disability Disadvantage: Experimental Evidence of Hiring Discrimination against Wheelchair Users. Eur Sociol Rev. 2021:37(5)818-33. https://doi.org/10.1093/esr/jcab004
Blonk L, Huijben T, Bredewold F, Tonkens E. Balancing care and work: a case study of recognition in a social enterprise. Disabil Soc. 2020:35(6):972-92. https://doi.org/10.1080/09687599.2019.1669434
Borghouts-van de Pas I, Freese C. Offering jobs to persons with disabilities: A Dutch employers' perspective. Alter. 2021:15(1):89-98. https://doi.org/10.1016/j.alter.2020.10.002
Bruyère S, Erickson W, VanLooy S. The impact of business size on employer ADA response. Rehabil Couns Bull. 2006:49(4):194-206. https://doi.org/10.1177/00343552060490040101
Brzykcy A, Boehm S. No such thing as a free ride: The impact of disability labels on relationship building at work. Hum Relat. 2022:75(4):734-63. https://doi.org/10.1177/0018726721991609

Buhariwala P, Wilton R, Evans J. Social enterprises as enabling workplaces for people with psychiatric disabilities. Disabil Soc. 2015:30(6):865-79. https://doi.org/10.1080/09687599.2015.1057318
Butterworth J, Hagner D. Workplace culture, social interactions, and supports for transition-age young adults. Ment Retard. 2000:38(4):342-53. https://doi.org/10.1352/0047-6765(2000)038<0342:WCSIAS>2.0.CO;2
Buys N, Rennie J. Developing relationships between vocational rehabilitation agencies and employers. Rehabil Couns Bull. 2001:44(2):95-103. https://doi.org/10.1177/003435520104400206
Carr D, Namkung E. Physical Disability at Work: How Functional Limitation Affects Perceived Discrimination and Interpersonal Relationships in the Workplace. J Hlth Soc Behav. 2021:62(4):545-61. https://doi.org/10.1177/00221465211023424
Carrier, S. Understanding social and professional integration as an adjustment process: Contribution to a theory of coadaptation. Int Dev Disabil. 2007:45(1):10-22. https://doi.org/10.1177/00221465211023424
Cavanagh J, Meacham H, Pariona-Cabrera P, Bartram T. Subtle workplace discrimination inhibiting workers with intellectual disability from thriving at the workplace. Pers Rev. 2021:50(7-8):1739-756. https://doi.org/10.1108/PR-10-2021-0723
Chan F, Strauser D, Maher P, Lee E, Jones R, Johnson E. Demand-side factors related to employment of people with disabilities: A survey of employers in the Midwest region of the United States. J Occup Rehabil. 2010:20:412-9. https://doi.org/10.1007/s10926-010-9252-6
Chan F, Tansey T, Iwanaga K, Bezyak J, Wehman P, Phillips B, Strauser D, Anderson D. Company characteristics, disability inclusion practices, and employment of people with disabilities in the post COVID-19 job economy: A cross sectional survey study. J Occup Rehabil. 2021:31:463-73. https://doi.org/10.1007/s10926-020-09941-8
Chandola T, Rouxel P. The role of workplace accommodations in explaining the disability employment gap in the UK. Soc Sci Med. 2021:285:114313. https://doi.org/10.1016/j.socscimed.2021.114313
Chen A, O'Neill J, Phillips K, Houtenville A, Katz E. Relationship of business practices and characteristics to supervisors' perceived effectiveness of disability recruitment. J Vocat Rehabil. 2023:59(3):301-10. https://doi.org/10.3233/JVR-230047
Chordiya R. Organizational inclusion and turnover intentions of federal employees with disabilities. Rev Public Pers Admin. 2020:42(1):60-87. https://doi.org/10.1177/0734371X20942305
Chow C, Cichocki B. Predictors of job accommodations for individuals with psychiatric disabilities. Rehabil Couns Bull. 2016-59(3):172-84. https://doi.org/10.1177/0034355215583057
Chow C, Cichocki B, Croft B. The impact of job accommodations on employment outcomes among individuals with psychiatric disabilities. Psychiat Serv. 2014:65(9):1126-33. https://doi.org/10.1176/appi.ps.201300267
Chowdhury D, Lund E, Carey C, Li Q. (2022). Intersection of Discriminations: Experiences of Women With Disabilities With Advanced Degrees in Professional Sector in the United States. Rehabil Psychol. 2022:67(1):28-41. https://doi.org/10.1037/rep0000419
Chumo I, Kabaria C, Mberu B. Social inclusion of persons with disability in employment: what would it take to socially support employed persons with disability in the labor market? Front Rehabil Sci. 2023:4. https://doi.org/10.3389/fresc.2023.1125129
Coll C, Mignonac K. Perceived organizational support and task performance of employees with disabilities: a need satisfaction and social identity perspectives. Int J Hum Resour Manag. 2023:34(10):2039-73. https://doi.org/10.1080/09585192.2022.2054284

Dalgin R, Bellini J. Invisible disability disclosure in an employment interview. Impact on employers' hiring decisions and views of employability. Rehabil Couns Bull. 2008:52(1):6-15. https://doi.org/10.1177/0034355207311311
Davies J, Heasman B, Livesey A, Walker A, Pellicano E, Remington A. Access to employment: A comparison of autistic, neurodivergent and neurotypical adults' experiences of hiring processes in the United Kingdom. Autism. 2023:27(6):1746-17. https://doi.org/10.1177/13623613221145377
de Carvalho-Freitas M, de Oliveira M, Tette R, Santos J. Organizational context and inclusion: Perceptions of managers and people with disabilities. Appl Psychol Int Rev. 2023. https://doi.org/10.1111/apps.12486
de Carvalho-Freitas M, Stathi S. Reducing workplace bias toward people with disabilities with the use of imagined contact. J Appl Soc Psychol. 2017:47(5):256-66. https://doi.org/10.1111/jasp.12435
Di Francesco C, Murahara F, Martin V, Flanagan T, Nadig A. The value of employment support services for adults on the autism spectrum and/or with intellectual disabilities: Employee, employer, and job coach perspectives. J Vocat Rehabil. 2021:55(3):283-96. https://doi.org/10.3233/JVR-211163
Dong S, Eto O, Spitz C. Barriers and facilitators to requesting accommodation among individuals with psychiatric disabilities: A qualitative approach. J Voc Rehab. 2021:55(2):207-218. https://doi.org/10.3233/JVR-211157
Eissenstat S, Lee Y, Hong S. An Examination of Barriers and Facilitators of Job Satisfaction and Job Tenure Among Persons With Disability in South Korea. Rehabil Couns Bull. 2022:65(4):310-21. https://doi.org/10.1177/00343552211006767
Erickson W, von Schrader S, Bruyere S, VanLooy S. The employment environment: Employer perspectives, policies, and practices regarding the employment of persons with disabilities. Rehabil Couns Bull. 2013:57(4):195-208. https://doi.org/10.1177/0034355213509841
Farris B, Stancliffe R. The co-worker training model: outcomes of an open employment pilot project. J Intellect Dev Dis. 2001:26(2):143-59. https://doi.org/10.1080/13668250020054459
Fillary R, Pernice R. Social inclusion in workplaces where people with intellectual disabilities are employed: implications for supported employment professionals. Int J Rehabil Res. 2006:29(1):31-7. https://doi.org/10.1097/01.mrr.0000185952.87304.63
Flores, N., Moret-Tatay, C., Gutierrez-Bermejo, B., Vazquez, A., & Jenaro, C. Assessment of Occupational Health and Job Satisfaction in Workers with Intellectual Disability: A Job Demands-Resources Perspective. Int J Env Res Pub Hlth. 2021:18(4):2072. https://doi/org/10.3390/ijerph18042072
Foster D, Fosh P. Negotiating 'difference': Representing disabled employees in the British workplace. Brit J Ind Relat. 2010:48(3):560-82. https://doi.org/10.1111/j.1467-8543.2009.00748.x
Garrels V, Sigstad H, Wenbelborg C, Dean E. Work opportunities and workplace characteristics for employees with intellectual disability in the Norwegian labour market. Int J Disabil Dev Ed. 2022. https://doi.org/10.1080/1034912X.2022.2150838
Gignac M, Shahidi F, Jetha A, Kristman V, Bowring J, Cameron J, Tonima S, Ibrahim S. Impacts of the COVID-19 pandemic on health, financial worries, and perceived organizational support among people living with disabilities in Canada. Disabil Hlth J. 2021:14(4). https://doi.org/ 10.1016/j.dhjo.2021.101161
Gignac M, Jetha A, Ginis K, Ibrahim S. Does it matter what your reasons are when deciding to disclose (or not disclose) a disability at work? The association of workers' approach and avoidance goals with perceived positive and negative workplace outcomes. J Occup Rehabil. 2021:31(3):638-51. https://doi.org/10.1007/s10926-020-09956-1

Gignac M, Bowring J, Jetha A, Beaton D, Breslin F, Franche R, Irvin E, Macdermid J, Shaw W, Smith P, Thompson A, Tompa E, Van Eerd D, Saunders R. Disclosure, privacy and workplace accommodation of episodic disabilities: Organizational perspectives on disability communication-support processes to sustain employment. J Occup Rehabil. 2021:31:153-65. https://doi.org/10.1007/s10926-020-09901-2
Gilbride D, Stensrud R, Ehlers C, Evans E, Peterson C. Employers' attitudes toward hiring persons with disabilities and Vocational Rehabilitation services. J Rehabil. 2000:66(4):17-24. https://doi.org/10.1017/jrc.2013.2
Gilbride D, Stensrud R, Vandergoot D, Golden K. Identification of the characteristics of work environments and employers open to hiring and accommodating people with disabilities. Rehabil Couns Bull. 2003:46(3):130-37. https://doi.org/10.1177/00343552030460030101
Gould R, Mullin C, Harris S, Jones R. Building, sustaining and growing: disability inclusion in business. Equal Divers Incl. 2022:41(3):418-34. https://doi.org/10.1108/EDI-06-2020-0156
Gray D, Morgan K, Gottlieb M, Hollingsworth H. Person factors and work environments of workers who use mobility devices. Work. 2014:48:349-59. https://doi.org/10.3233/WOR-141907
Gröschl S. Presumed incapable: Exploring the validity of negative judgments about persons with disabilities and their employability in hotel operations. Cornell Hosp Q. 2012:54(2):114-23. https://doi.org/10.1177/1938965512453082
Gröschl S. An exploration of HR policies and practices affecting the integration of persons with disabilities in the hotel industry in major Canadian tourism destinations. Int J Hosp Manag. 2007:26(3):666-86. https://doi.org/10.1016/j.ijhm.2006.05.007
Grijseels M, Zuiderent-Jerak T, Regeer B. Technologies for inclusive employment: beyond the prosthetic fix-social transformation axis. Disabil Soc. 2023:38(9):1534-57. https://doi.org/10.1080/09687599.2021.1997720
Grzeskowiak A, Zaluska U, Kwiatkowska-Ciotucha D, Kozyra C. People with disabilities in the workplace: Results of a survey conducted among Polish and Finnish employers. Int J Env Res Public Hlth. 2021:18(20):10934. https://doi.org/10.3390/ijerph182010934
Guillaume M, Loufrani-Fedida, S. Stakeholder engagement in inclusive employability management for employees whose health at work is impaired: empirical evidence from a French public organisation. Pers Rev. 2022:52(1):121-44. https://doi.org/10.1108/PR-06-2021-0404
Haafkens J, Kopnina H, Meerman M, van Dijk F. Facilitating job retention for chronically ill employees: perspectives of line managers and human resource managers. Bmc Hlth Serv Res. 2011:11: 104. https://doi.org/10.1186/1472-6963-11-104
Habeck R, Hunt A, Rachel C, Kregel J, Chan F. Employee retention and integrated disability management practices as demand side factors. J Occup Rehabil. 2010:20:443-55. https://doi.org/10.1007/s10926-009-9225-9
Hagner D, Dague B, Phillips K. Including employees with disabilities in workplace cultures: Strategies and barriers. Rehabil Couns Bull. 2015:58(4):195-202. https://doi.org/10.1177/0034355214544750
Hemphill E Kulik C. Which employers offer hope for mainstream job opportunities for disabled people? Soc Pol Soc. 2016a:15(4):537-54. https://doi.org/10.1017/S1474746415000457
Hemphill E, Kulik C. Shaping attitudes to disability employment with a national disability insurance scheme. Aust J Soc Issues. 2016b:51(3):299-317. https://doi.org/10.1002/j.1839-4655.2016.tb01233.x
Ho J, Bonaccio S, Connelly C, Gellatly I. Representative-negotiated i-deals for people with disabilities. Hum Resour Manag. 2022:61(6):681-98. https://doi.org/10.1002/hrm.22118

Houtenville A, Kalargyrou V. People with disabilities: Employers' perspectives on recruitment practices, strategies, and challenges in leisure and hospitality. Cornell Hosp Q. 2012:53(1), 40-52. https://doi.org/10.1177/1938965511424151
Houtenville A, Kalargyrou V. Employers' perspectives about employing people with disabilities: A comparative study across industries. Cornell Hosp Q. 2015:56(2): 168-79. https://doi.org/10.1177/1938965514551633
Huang I, Chen R. Employing people with disabilities in the Taiwanese workplace: Employers' perceptions and considerations. Rehabil Couns Bull. 2015:59(1):43-54. https://doi.org/10.1177/0034355214558938
Ishii K, Yaeda J. Job development activities for individuals with intellectual disabilities in Japan. J Rehabil. 2010:76(2):11-6.
Jammaers E. Theorizing discursive resistance to organizational ethics of care through a multi-stakeholder perspective on disability inclusion practices. J Bus Ethics. 2022:183:333-345. https://doi.org/10.1007/s10551-022-05079-0
Jansson I, Bjorklund A, Perseius K, Gunnarsson A. The concept of 'work ability' from the view point of employers. Work, 2015:52:153-67. https://doi.org/10.3233/WOR-152037
Johnson T, Joshi A, Kreiner G. Bridgework: A model of brokering relationships across social boundaries in organizations. Organ Sci. 2023:34(4):1458-86. https://doi.org/10.1287/orsc.2022.1631
Karl M, Pegg S, Harpur P. Exploring constraints in business travel for disabled workers: An ecological systems perspective. Disabil Soc. 2022. https://doi.org/10.1080/09687599.2022.2088333
Kensbock J, Boehm S. The role of transformational leadership in the mental health and job performance of employees with disabilities. Int J Hum Resour Manag. 2016:27(14):1580-609. https://doi.org/10.1080/09585192.2015.1079231
Kosyluk K, Corrigan R. Employer stigma as a mediator between past and future hiring behavior. Rehab Couns Bull. 2014:57(2):102-8. https://doi.org/10.1177/0034355213496284
Krogh C. The relationality of workplace accessibility - employers' perceptions of accessibility and the impact on recruitment of wheelchair users. Disabil Soc. 2023. https://doi.org/10.1080/09687599.2023.2254471
Kulkarni M. Hiding but hoping to be found: workplace disclosure dilemmas of individuals with hidden disabilities. Equal Divers Incl, 2022:41(3):491-507. https://doi.org/10.1108/EDI-06-2020-0146
Kwan C. Helping People with disabilities in the workplace: Mezzo-level interventions targeting corporate culture. Soc Work. 2021:66(4):339-47. https://doi.org/10.1093/sw/swab030
L'Horty Y, Mahmoudi N, Petit P, Wolff F.. Is disability more discriminatory in hiring than ethnicity, address or gender? Evidence from a multi-criteria correspondence experiment. Soc Sci Med. 2022:303:114990. https://doi.org/10.1016/j.socscimed.2022.114990
Lindsay S, Adams T, Sanford R, McDougall C, Kingsnorth S, Menna-Dack D. Employers' and employment counselors' perceptions of desirable skills for entry-level positions for adolescents: how does it differ for youth with disabilities? *Disability & Society*, 2014:29(6):953-67. https://hdl.handle.net/1807/110492
Lundberg C. Striving to abolish a deficit approach to disability: frames applied by frontline workers and activist entrepreneurs in employment. Disabil Soc. 2022. https://doi.org/10.1080/09687599.2022.2160927
Luu T. Engaging employees with disabilities in Vietnamese business context: The roles of disability inclusive HR practices and mediation and moderation mechanisms. Empl Relat. 2018:40(5):822-47. https://doi.org/10.1108/ER-06-2017-0134

Lyubykh Z, Ansari M, Williams-Whitt K, Kristman V. Disability severity, leader-member exchange, and attitudinal outcomes: Considering the employee and supervisor perspectives. J Occup Rehabil. 2020:30:613-23. https://doi.org/10.1007/s10926-020-09884-0
Maddison J, Brooks J, Graham K, Birks Y. 'They exist but they don't exist': Personal Assistants supporting physically disabled people in the workplace. Work Empl Soc. 2022:37(4):1052-69. https://doi.org/10.1177/09500170221075532
Mai V, Thu N, Thu L, Nguyen P. Blue Ocean strategy as inclusive innovation policy for implementing disability employment legal rules from Vietnamese enterprise perspective. Lex Hum. 2022:14(2):208-30. https://www.proquest.com/scholarly-journals/blue-ocean-strategy-as-inclusive-innovation/docview/2729114590/se-2
Man, X, Zhu X, Sun C. The positive effect of workplace accommodation on creative performance of employees with and without disabilities. Front Psychol. 2020:11:1217. https://doi.org/10.3389/fpsyg.2020.01217
Mank D, Cioff A, Yovanoff P. Direct support in Supported Employment and its relation to job typicalness, coworker involvement, and employment outcomes. Ment Retard. 2000:38(6):506-16. https://doi.org/10.1352/0047-6765(2000)038<0506:DSISEA>2.0.CO;2
McDonnall M. Factors associated with employer hiring decisions regarding people who are blind or have low vision. J Visual Impair Blin. 2018:112(2):197-204. https://doi.org/10.1177/0145482X1811200207
McDonough J, Ham W, Brooke A, Wehman P, Wright T, Godwin J, Junod P, Hurst R. Health care executive perceptions of hiring and retention practices of people with disabilities: Results from executive focus groups. Rehabil Couns Bull. 2021:64(2):75-85. https://doi.org/10.1177/0034355220915766
McKinney E, Swartz L. Employment integration barriers: experiences of people with disabilities. Int J Hum Resour Manag. 2021:32(10):2298-320. https://doi.org/10.1080/09585192.2019.157974
Meacham H, Cavanagh J, Shaw A, Bartram T. Innovation programs at the workplace for workers with an intellectual disability. Two case studies in large Australian organisations. Pers Rev. 2017a:46(7):1381-1396. https://doi.org/10.1108/PR-08-2016-0214
Meacham H, Cavanagh J, Shaw A, Bartram T. HRM practices that support the employment and social inclusion of workers with an intellectual disability. Pers Rev. 2017b:46(8): 1475-92. https://doi.org/10.1108/PR-05-2016-0105
Michna A, Kmieciak R, Burzynska-Ptaszek K. Job preferences and expectations of disabled people and small and medium-sized enterprises in Poland: Implications for disabled people's professional development. Hum Resour Dev Q. 2017:28(3):299-337. https://doi.org/10.1002/hrdq.21280
Miller L, Gottlieb M, Morgan K, Gray D. Interviews with employed people with mobility impairments and limitations: Environmental supports impacting work acquisition and satisfaction. Work. 2014:48(3):361-72. https://doi.org/10.3233/WOR-131784
Moody L, Saunders J, Leber M, Wojcik-Augustyniak W, Szajczyk M, Rebernik N. An exploratory study of barriers to inclusion in the European workplace. Disabil Rehabil. 2017:39(20):2047-54. https://doi.org/10.1080/09638288.2016.1217072
Mousa M, Samara G. The institutional limitations of emancipation: The inclusion of disabled employees in the Egyptian public context post COVID-19. Int J Public Admin. 2022:46(13): 939-50. https://doi.org/10.1080/01900692.2022.2049815
Munsell E, Kudla A, Su H, Wong J, Crown D, Capraro P, Trierweiler R., Park M, Heinemann A. Employers' perceptions of challenges and strategies in hiring, retaining, and promoting employees with physical disabilities. Rehabil Couns Bull. 2022. https://doi.org/10.1177/00343552221130304

Nelissen P, Hulsheger U, van Ruitenbeek G, Zijlstra F. How and when stereotypes relate to inclusive behavior toward people with disabilities. Int J Hum Resour Manag. 2016:27(14):1610-25. https://doi.org/10.1080/09585192.2015.1072105
Nota L, Santilli S, Ginevra M, Soresi S. Employer attitudes towards the work inclusion of people with disability. J Appl Res Intellect Disabil. 2014:27:511-20. https://doi.org/10.1111/jar.12081
Novak J, Rogan P. Social integration in employment settings: Application of intergroup contact theory. Intel Dev Disabil. 2010:18(1):31-51. htpps://doi.org/10.1352/1934-9556-48.1.31
Olsen J. Employers: influencing disabled people's employment through responses to reasonable adjustments. Disabil Soc. 2022. https://doi.org/10.1080/09687599.2022.2099251
Østerud K. A balancing act: The employer perspective on disability disclosure in hiring. J of Vocat Rehabil. 2022:56(3):289-302. https://doi.org/10.3233/JVR-221192
Østerud K, Vedeler J. Disability and regulatory approaches to employer engagement: Cross-national challenges in bridging the gap between motivation and hiring practice. Soc Pol Soc. 2022. https://doi.org/10.1017/S1474746422000021
Paluch T, Fossey E, Harvey C. Social firms: Building cross-sectoral partnerships to create employment opportunity and supportive workplaces for people with mental illness. Work. 2012:43:63-75. https://doi.org/10.3233/WOR-2012-1448
Papakonstantinou D. Papadopoulos K. Social support in the workplace for working-age adults with visual impairments. J Visual Impair Blin. 2009:42(6):798-805. https://doi.org/10.1177/0145482X0910300703
Papakonstantinou D, Papadopoulos K. Employers' attitudes toward hiring individuals with visual impairments. Disabil Rehabil. 2020:42(6):798-805. htpps://doi.org/10.1080/09638288.2018.1510044
Perez-Conesa F, Romeo M, Yepes-Baldo M. Labour inclusion of people with disabilities in Spain: the effect of policies and human resource management systems. Int J Hum Resour Manag. 2020:31(6):785-804. https://doi.org/10.1080/09585192.2017.1380681
Pettersen K, Fugletveit R. "Should we talk about it?": A study of the experiences business leaders have of employing people with mental health problems. Work. 2015:52(3):635-41. https://doi.org/10.3233/WOR-152125
Richards J, Sang K. Trade unions as employment facilitators for disabled employees. Int J Hum Resour Manag. 2016:27(14):1642-61. https://doi.org/10.1080/09585192.2015.1126334
Richards J, Sang K, Marks A, Gill S. "I've found it extremely draining". Emotional labour and the lived experience of line managing neurodiversity. Pers Rev. 2019:48(7):1903-23. https://doi.org/10.1108/PR-08-2018-0289
Sanclemente F, Gamero N, Medina F, Mendoza-Denton R. A multilevel model of job inclusion of employees with disabilities: The role of organizational socialization tactics, coworkers social support, and an inclusive team context. Appl Psychol Int Rev. 2022 https://dx.doi.org/10.1111/apps.12390
Sang K, Calvard T, Remnant J. Disability and academic careers: Using the social relational model to reveal the role of Human Resource Management practices in creating disability. Work Empl Soc. 2022:36(4):722-40. https://doi.org/10.1177/0950017021993737
Schaap R, Coenen P, Zwinkels W, de Wolff M, Hazelzet A, Anema J. Training for supervisors to improve sustainable employment of employees with a work disability: A longitudinal effect and process evaluation from an intervention study with matched controls. J Occup Rehabil. 2023. https://doi.org/10.1007/s10926-023-10118-2
Schaap R, Stevels V, de Wolff M, Hazelzet A, Anema J, Coenen P. "I noticed that when I have a good supervisor, it can make a Lot of difference." A qualitative study on guidance of

employees with a work disability to improve sustainable employability. J Occup Rehabil. 2023:33(1):201-12. https://doi.org/10.1007/s10926-022-10063-6
Scheid T. Stigma as a barrier to employment: Mental disability and the Americans with Disabilities Act. Int J Law Psychiat. 2005:28(6):670-90. https://doi.org/10.1016/j.ijlp.2005.04.003
Schiffmann B, Finger M, Karcz M, Staubli S, Trezzini B. Factors related to sustainable employment of people with acquired brain injury or spinal cord injury: The employer's perspective. Front Rehabil Sci. 2022:3, https://doi.org/10.3389/fresc.2022.876389
Schreuer S, Myhill W, Aratan-Bergman T, Samant D, Blanck P. Workplace accommodations: Occupational therapists as mediators in the interactive process. Work. 2009:34(2):149-60. https://doi.org/10.3233/WOR-2009-0913
Schur L, Han K, Kim A, Ameri M, Blanck P, Kruse D. Disability at work: A look back and forward. J Occup Rehabil. 2017:27(4):482-97. https://doi.org/10.1007/s10926-017-9739-5
Schur L, Kruse D, Blasi J, Blanck P. Is disability disabling in all workplaces? Workplace disparities and corporate culture. Ind Relat. 2009:48(3):381-411. https://doi.org/10.1111/j.1468-232X.2009.00565.x
Schur L, Ameri M, Kruse D. Telework after COVID: A "silver lining" for workers with disabilities? J Occup Rehabil. 2020:30:521-36. https://doi.org/10.1007/s10926-020-09936-5
Sever M, Özdemir S. "Never hire those—!" Hiring stigma in Turkish organizations: An exploratory sequential mixed methods approach. Anal Soc Iss Pub Pol. 2021:21(1):708-33. https://doi.org/10.1111/asap.12239
Shahid N, Zahid G. Exploration of HR managers perspectives in hiring and retaining practices of people with physical disabilities. Pertanika J Soc Sci Hum. 2021:29(2):1071-90.
Shuey K, Jovic E. Disability accommodation in nonstandard and precarious employment arrangements. Work Occup. 2013:40(2):174-205. https://doi.org/10.1177/0730888413481030
Sibbald K, Beagan B. Disabled healthcare professionals' experiences of altruism: identity, professionalism, competence, and disclosure. Disabil Soc. 2022:39(1):174-91. https://doi.org/10.1080/09687599.2022.2061333
Simonsen M, Fabian E, Luecking R. Employer preferences in hiring youth with disabilities. J Rehabil. 2015:81(1):9-18.
Solovieva T, Dowler D, Walls R. Employer benefits from making workplace accommodations. Disabil Hlth J. 2011:4(1):39-45. https://doi.org/10.1016/j.dhjo.2010.03.001
Stevens G. Employers' perceptions and practice in the employability of disabled people: a survey of companies in south east UK. Disabil Soc. 2002:17(7):779-96. https://doi.org/10.1080/0968759022000039073
Strindlund L, Abrandt-Dahlgren M, Stahl D. Employers' views on disability, employability, and labor market inclusion: a phenomenographic study. Disabil Rehabil. 2019:41(24):2910-17. https://doi.org/10.1080/09638288.2018.1481150
Suresh V, Dyaram L. Diversity in disability: leaders' accounts on inclusive employment in the Indian context. Equal Div Incl. 2022:41(3), 454-473. https://doi.org/10.1108/EDI-05-2020-0133
Svinndal E, Jensen C, Rise M. (2020) Employees with hearing impairment. A qualitative study exploring managers' experiences. Disabil Rehabil. 2020:42(13):1855-62. https://doi.org/10.1080/09638288.2018.1541101
Toldra R, Santos M. (2013) People with disabilities in the labor market: Facilitators and barriers. Work. 2013:45:553-63. https://doi.org/10.3233/WOR-131641
Trezzini B, Schuller V, Schupbach S, Bickenbach J. Environmental barriers to and facilitators of labour market participation as experienced by disabled people living in Switzerland. Disabil Soc. 2021:36(6):925-51. https://doi.org/10.1080/09687599.2020.1768053

Tuan L, Rowley C, Khai D, Qian D, Masli E, Le H. Fostering well-being among public employees with disabilities: The roles of disability-inclusive human resource practices, job resources, and public service motivation. Rev Public Pers Admin. 2021:41(3):466-96. https://doi.org/10.1177/0734371X19897753
Ulstein J. The impact of employer characteristics on sustaining employment for workers with reduced capacity: Evidence from Norwegian register data. Soc Pol Soc. 2023. https://doi.org/10.1017/S1474746423000027
Uppal S. Disability, workplace characteristics and job satisfaction. In J Manpower. 2005:26(4):336-49. https://doi.org/10.1108/01437720510609537
van Berkel R. Employer engagement in promoting the labour-market participation of jobseekers with disabilities. An employer perspective. Soc Pol Soc. 2021:20(4):533-47. https://doi.org/10.1017/S147474642000038X
Vedeler J. How is disability addressed in a job interview? Disabil Soc. 2022. https://doi.org/10.1080/09687599.2022.2162860
Vedeler J, Schreuer N. Policy in action: Stories on the workplace accommodation process. J Disabil Pol Stu. 2011:22(2):95-105. https://doi.org/10.1177/1044207310395942
Villotti P, Corbiere M, Fossey E, Fraccaroli F, Lecomte T, Harvey C. Work accommodations and natural supports for employees with severe mental illness in social businesses: An international comparison. Community Ment Hlth J. 2017:53(7):864870. https://doi.org/10.1007/s10597-016-0068-5
Wehman P, Sima A, Iwanaga K, McDonough J, Brooke A, Godwin J, Brooke V. Organizational factors influencing coworkers' attitudes toward employees with disabilities: A hierarchical regression analysis. Rehabil Res Pol Ed. 2021:35(2):83-93. https://doi.org/10.1891/RE-20-22
Wen B, Van Rensburg H, O'Neill S, Attwood T. Autism in the Australian workplace: the employer perspective. Asia Pac J Hum Resour. 2022:61(1):146-67. https://doi.org/10.1111/1744-7941.12333
Wendelborg C, Garrels V, Sigstad H, Dean E. Recruitment and work arrangements for employees with intellectual disability in competitive employment. J Pol Pract Intel Disabil. 2022:19(4):350-59. https://doi.org/10.1111/jppi.12418
Westmorland M, Williams R, Amick B, Shannon H, Rasheed F. Disability management practices in Ontario workplaces: Employees' perceptions. Disabil Rehabil. 2005:27(14):825-35. https://doi.org/10.1080/09638280400020631
Wiggett-Barnard C, Swartz L. What facilitates the entry of persons with disabilities into South African companies? Disabil Rehabil. 2012:34(12):1016-23. https://doi.org/0.3109/09638288.2011.631679
Wilson-Kovacs D, Ryan M, Haslam S, Rabinovich A. “Just because you can get a wheelchair in the building doesn't necessarily mean that you can still participate”: barriers to the career advancement of disabled professionals. Disabil Soc. 2008:23(7):705-17. https://doi.org/10.1080/09687590802469198
Wilton R, Schuer S. Towards socio-spatial inclusion? Disabled people, neoliberalism and the contemporary labour market. Area. 2006:38(2):186-95. https://www.jstor.org/stable/20004525
Wolffe K, Candela A. A qualitative analysis of employers' experiences with visually impaired workers. J Visual Impair Blin. 2002:96(9):622-35. https://doi.org/10.1177/0145482X0209600903
Woodhams C, Corby S. Then and now: Disability legislation and employers' practices in the UK. Brit J Ind Relat. 2007:45(3):556-80. https://doi.org/10.1111/j.1467-8543.2007.00628.x
Zhu X, Law K, Sun C, Yang D. Thriving of employees with disabilities: The roles of job self-efficacy, inclusion, and team-learning climate. Hum Resour Manag. 2019:58(1):21-34. https://doi.org/10.1002/hrm.21920
