# Supplementary material for: Organizational Practices for the Inclusion of People with Disabilities. A Scoping Review
Source: J Occup Rehabil. 2024 Jul 30;35(3):469–78. doi: 10.1007/s10926-024-10228-5 (PMC12361267; doi:10.1007/s10926-024-10228-5)
Supplement: Supplementary file 3 — Supplementary file3 (PDF 183 kb) [file 10926_2024_10228_MOESM3_ESM.pdf]

ORGANIZATIONAL PRACTICES FOR THE INCLUSION OF PEOPLE WITH DISABILITIES
Journal of Occupational Rehabilitation
Rik van Berkel, Eric Breit
r.vanberkel@uu.nl

Appendix 3. Review articles and some core characteristics

	Methods	Country of data collection	Content group*
Ameri, Kruse et al., 2023	Quantitative	USA	6
Ameri, Kurtzberg et al., 2023	Mixed	USA	3
Araten-Bergman, 2016	Quantitative	Israël	1, 2
Baldwin et al., 2014	Quantitative	Australia	6
Banks et al., 2001	Quantitative	USA	4
Bartram et al., 2021	Qualitative	Australia	4
Becerra et al., 2018	Qualitative	Spain	4
Berre, 2023	Quantitative	Norway	2
Bishop et al., 2007	Quantitative	USA	3
Bjørnshagen, 2022	Quantitative	Norway	2
Bjørnshagen et al., 2021	Quantitative	Norway	2
Blonk et al., 2020	Qualitative	Netherlands	4
Borghouts-van de Pas & Freese, 2021	Qualitative	Netherlands	3
Bruyère et al., 2006	Quantitative	USA	5
Brzykcy & Boehm, 2022	Quantitative	Germany	5
Buhariwala et al., 2015	Qualitative	Canada	6
Butterworth & Hagner, 2000	Qualitative	USA	4
Buys & Rennie, 2001	Qualitative	Australia	5
Carr & Namkung, 2021	Quantitative	USA	5
Carrier, 2007	Qualitative	USA	4
Cavanagh et al., 2021	Qualitative	Australia	4
Chan et al., 2010	Quantitative	USA	2

Chan et al., 2021	Quantitative	USA	1
Chandola & Rouxel, 2021	Quantitative	UK	4
Chen et al., 2023	Quantitative	USA	3
Chordiya, 2020	Quantitative	USA	4
Chow & Cichocki, 2016	Quantitative	USA	5
Chow et al., 2014	Quantitative	USA	4
Chowdury et al., 2022	Qualitative	USA	4
Chumo et al., 2023	Qualitative	Kenya	5
Coll & Mignonac, 2023	Quantitative	France	4
Dalgin & Bellini, 2008	Quantitative	USA	2
Davies et al., 2023	Quantitative	UK	3
de Carvalho-Freitas et al., 2023	Quantitative	Brazil	3, 4
de Carvalho-Freitas et al., 2017	Quantitative	Brazil	4
Di Francesco et al., 2021	Quantitative	Canada	6
Dong et al., 2021	Qualitative	USA	5
Eissenstat et al., 2022	Quantitative	South Korea	4
Erickson et al., 2013	Quantitative	USA	5
Farris & Stancliffe, 2001	Quantitative	USA	4
Fillary & Pernice, 2006	Qualitative	New Zealand	4
Flores et al., 2021	Quantitative	Spain	4
Foster & Fosh, 2010	Qualitative	UK	4
Garrels et al., 2022	Quantitative	Norway	1, 6
Gignac, Shahidi et al., 2021	Quantitative	Canada	5
Gignac, Jetha et al., 2021	Quantitative	Canada	5
Gignac, Bowring et al., 2021	Qualitative	Canada	4
Gilbride et al., 2000	Quantitative	USA	3
Gilbride et al., 2003	Qualitative	USA	6
Gould et al., 2022	Qualitative	USA	6
Gray et al., 2014	Quantitative	USA	4
Gröschl, 2012	Qualitative	Germany	6
Gröschl, 2007	Qualitative	Canada	3, 6

Grijseels et al., 2023	Qualitative	Netherlands	4
Grzeskowiak et al., 2021	Quantitative	Poland, Finland	1
Guillaume & Loufrani-Fedida, 2022	Qualitative	France	4
Haafkens et al., 2011	Qualitative	Netherlands	4
Habeck et al., 2010	Quantitative	USA	1, 4
Hagner et al., 2015	Quantitative	USA	6
Hemphill & Kulik, 2016a	Qualitative	Australia	1
Hemphill & Kulik, 2016b	Quantitative	Australia	1
Ho et al., 2022	Qualitative	Canada	5
Houtenville & Kalargyrou, 2012	Quantitative	USA	2, 3
Houtenville & Kalargyrou, 2015	Quantitative	USA	1, 2, 3
Huang & Chen, 2015	Qualitative	Taiwan	3, 6
Ishii & Yaeda, 2010	Quantitative	Japan	4
Jammaers, 2022	Qualitative	Belgium	5
Jansson et al., 2015	Qualitative	Sweden	6
Johnson et al., 2023	Qualitative	USA	4
Karl et al., 2022	Qualitative	Australia	4
Kensbock & Boehm, 2016	Mixed	Israel	4
Kosyluk & Corrigan, 2014	Quantitative	USA	2
Krogh, 2023	Qualitative	Denmark	3
Kulkarni, 2022	Qualitative	India	4
Kwan, 2021	Qualitative	China	4
L'Horty et al., 2022	Quantitative	France	2
Lindsay et al., 2014	Qualitative	Canada	3
Lundberg, 2022	Qualitative	Norway	3
Luu, 2018	Quantitative	Vietnam	4
Lyubykh et al., 2020	Quantitative	USA	4
Maddison et al., 2022	Qualitative	UK	6
Mai et al., 2022	Qualitative	Vietnam	6
Man et al., 2020	Quantitative	China	4
Mank et al., 2000	Quantitative	USA	4

McDonnall, 2018	Quantitative	USA	1
McDonough et al., 2021	Qualitative	USA	6
McKinney & Schwartz, 2021	Qualitative	South Africa	3
Meacham et al., 2017a	Qualitative	Australia	4
Meacham et al., 2017b	Qualitative	Australia	4
Michna et al., 2017	Mixed	Poland	3
Miller et al., 2014	Qualitative	USA	6
Moody et al.,, 2017	Quantitative	Uk, Spain, Italy, Poland, Slovenia, Belgium	5
Mousa & Samara, 2022	Qualitative	Egypt	4
Munsell et al., 2022	Quantitative	USA	3, 6
Nelissen et al., 2016	Quantitative	Netherlands	4
Nota et al., 2014	Quantitative	Italy	2
Novak & Rogan, 2010	Quantitative	USA	4
Olsen, 2022	Qualitative	UK	6
Østerud, 2022	Qualitative	Norway	6
Østerud & Vedeler, 2022	Qualitative	USA, Norway	3
Paluch et al., 2012	Qualitative	Australia	4
Papakonstantinou & Papadopoulos, 2009	Qualitative	Greece	6
Papakonstantinou & Papadopoulos, 2020	Quantitative	Greece	2
Perez-Conesa et al., 2020	Quantitative	Spain	1
Pettersen & Fugletveit, 2015	Qualitative	Norway	3
Richards et al., 2016	Qualitative	UK	5
Richards et al., 2019	Qualitative	UK	5
Sanclemente et al., 2022	Quantitative	Spain	4
Sang et al., 2022	Qualitative	UK	6
Schaap, Coenen et al., 2023	Quantitative	Netherlands	4
Schaap, Stevels et al., 2023	Qualitative	Netherlands	4
Scheid, 2005	Quantitative	USA	1, 2
Schiffmann et al., 2022	Qualitative	Switzerland	4

Schreuer et al., 2009	Qualitative	USA	5
Schur et al., 2017	Quantitative	USA	4
Schur et al., 2009	Quantitative	USA	4
Schur et al., 2020	Quantitative	USA	4
Sever & Özdemir, 2021	Mixed	Turkey	3
Shahid & Zahid, 2021	Qualitative	Pakistan	3
Shuey & Jovic, 2013	Quantitative	Canada	4
Sibbald & Beagan, 2022	Qualitative	Canada	4
Simonsen et al., 2015	Quantitative	USA	3
Solovieva et al., 2011	Quantitative	USA	4
Stevens, 2002	Quantitative	UK	3
Strindlund, 2019	Qualitative	Sweden	3
Suresh & Dyaram, 2022	Qualitative	India	3
Svinndal et al., 2020	Qualitative	Norway	6
Toldra & Santos, 2013	Qualitative	Brazil	6
Trezzini et al., 2021	Qualitative	Switzerland	4
Tuan et al., 2021	Qualitative	Vietnam	4
Ulstein, 2023	Quantitative	Norway	4
Uppal, 2005	Quantitative	Canada	4
Van Berkel, 2021	Mixed	Netherlands	6
Vedeler, 2022	Qualitative	Norway	3
Vedeler & Schreuer, 2011	Qualitative	USA, Norway	5
Villotti et al., 2017	Quantitative	Canada	4
Wehman et al., 2021	Quantitative	USA	4
Wen et al., 2022	Qualitative	Australia	6
Wendelborg et al., 2022	Quantitative	Norway	3, 6
Westmorland et al., 2005	Mixed	Canada	6
Wiggett-Barnard & Schwartz, 2012	Quantitative	South Africa	2
Wilson-Kovacs et al., 2008	Qualitative	UK	4
Wilton & Schuer, 2006	Qualitative	Canada	3
Wolffe & Candela, 2002	Qualitative	USA	3, 6

Woodhams & Corby, 2007	Quantitative	UK	1
Zhu et al., 2019	Quantitative	China	4

* The core focus of the study is: 1=identifying factors related to actual hiring; 2=identifying factors related to intention/likelihood to hire; 3=exploring factors relevant for hiring processes; 4=outcomes of organizational practices aimed at the inclusion of employees with disabilities; 5=conditions under which organizational practices seen as contributing to inclusion of employees with disabilities can be implemented; 6=exploring practices implemented in hiring organizations aimed at including employees with disabilities.
