# Supplementary material for: Organizational Practices for the Inclusion of People with Disabilities. A Scoping Review
Source: J Occup Rehabil. 2024 Jul 30;35(3):469–78. doi: 10.1007/s10926-024-10228-5 (PMC12361267; doi:10.1007/s10926-024-10228-5)
Supplement: Supplementary file 4 — Supplementary file4 (PDF 126 kb) [file 10926_2024_10228_MOESM4_ESM.pdf]

ORGANIZATIONAL PRACTICES FOR THE INCLUSION OF PEOPLE WITH DISABILITIES
Journal of Occupational Rehabilitation
Rik van Berkel, Eric Breit
r.vanberkel@uu.nl

Appendix 4. Categorization of journals

Category	Journal title
Social policy/Social work studies	Analyses of Social Issues and Public Policy Journal of Disability Policy Studies<br>Social Policy & Society<br>Social Work
Organization/Work/HRM studies	Asia Pacific Journal of Human Resources<br>British Journal of Industrial Relations<br>Cornell Hospitality Journal<br>Employee Relations<br>Equality Diversity and Inclusion<br>Human Relations<br>Human Resource Development Quarterly<br>Human Resource Management<br>Industrial Relations<br>International Journal of Hospitality<br>International Journal of Human Resource Management<br>International Journal of Manpower<br>Journal of Business Ethics<br>Management (IJHM), Review of Public<br>Organization Science<br>Personnel Administration<br>Personnel Review<br>Work Employment and Society<br>Work and Occupations
Rehabilitation studies	Disability and Rehabilitation<br>Frontiers in Rehabilitation Sciences<br>International Journal of Rehabilitation Research<br>Journal of Rehabilitation<br>Journal of Occupational Rehabilitation<br>Journal of Vocational Rehabilitation<br>Psychiatric Rehabilitation Journal<br>Rehabilitation Counseling Bulletin<br>Rehabilitation Psychology<br>Rehabilitation Research, Policy and Education<br>Work – A journal of Prevention Assessment & Rehabilitation
Disability studies	Alter<br>Autism<br>Disability and Health Journal<br>Disability & Society<br>Epilepsy and Behavior<br>Intellectual and Developmental Disabilities<br>International Journal of Disability Development and Education

	Journal of Applied Research in Intellectual Disabilities<br>Journal of Developmental and Autism Disorders<br>Journal of Intellectual & Developmental Disability<br>Journal of Policy and Practice in Intellectual Disabilities<br>Journal of Visual Impairment and Blindness<br>Mental Retardation
Health/Medicine studies	Bmc Health Services Research<br>Community Mental Health Journal<br>International Journal of Environmental Research and Public Health<br>Journal of Health and Social Behavior<br>Social Science & Medicine
Other	Applied Psychology – an International Review<br>Area<br>Australian Journal of Social Issues<br>European Sociological Review<br>Frontiers in Psychology<br>International Journal of Law & Psychiatry<br>International Journal of Public Administration<br>Journal of Applied Social Psychology<br>Lex Humana<br>Pertanika Journal of Social Science and Humanities<br>Psychiatric Services<br>Social Sciences & Humanities
